# Supplementary material for: A gene expression map of shoot domains reveals regulatory mechanisms
Source: Nat Commun. 2019 Jan 11;10:141. doi: 10.1038/s41467-018-08083-z (PMC6329838; doi:10.1038/s41467-018-08083-z)
Supplement: Supplementary file 1 — Supplementary Information [file 41467_2018_8083_MOESM1_ESM.pdf]

SUPPLEMENTARY FIGURES

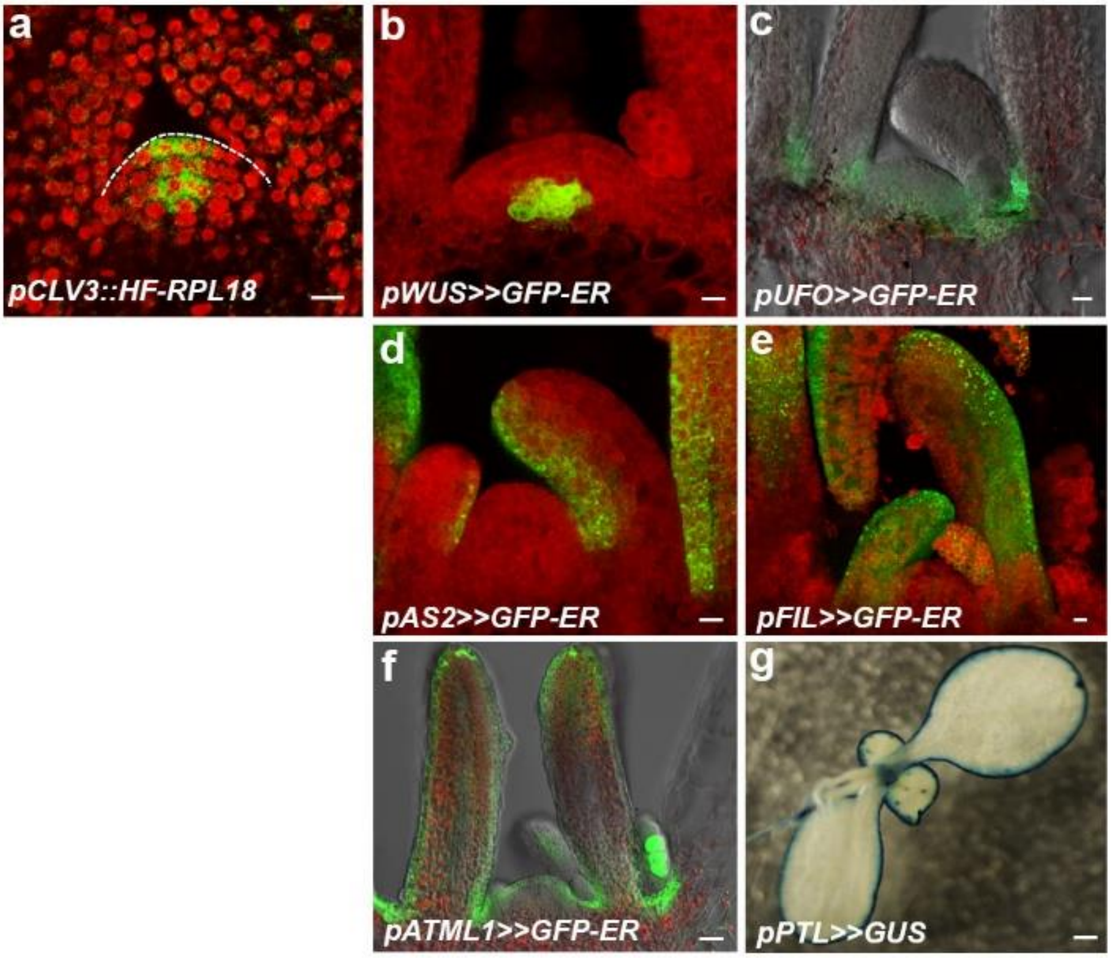

Supplementary Figure 1. Domain-specific promoter activities, related to Figure 1.

(**a**-**f**) Immunolocalization of *pCLV3::HF-RPL18* (**a**), and expression of *pWUS>>GFP-ER* (**b**), *pUFO>>GFP-ER* (**c**), *pAS2>>GFP-ER* (**d**), *pFIL>>GFP-ER* (**e**) and *pATML1>>GFP-ER* (**f**) in longitudinal sections through vegetative shoot apex of 7 DAG wild-type like plants. Bars = 10µm. (**g**) Pattern of GUS expression in a *pPTL>>GUS* plant displaying leaf marginal expression. Bars = 500µm. A dotted line in (**a**) indicates the meristem.

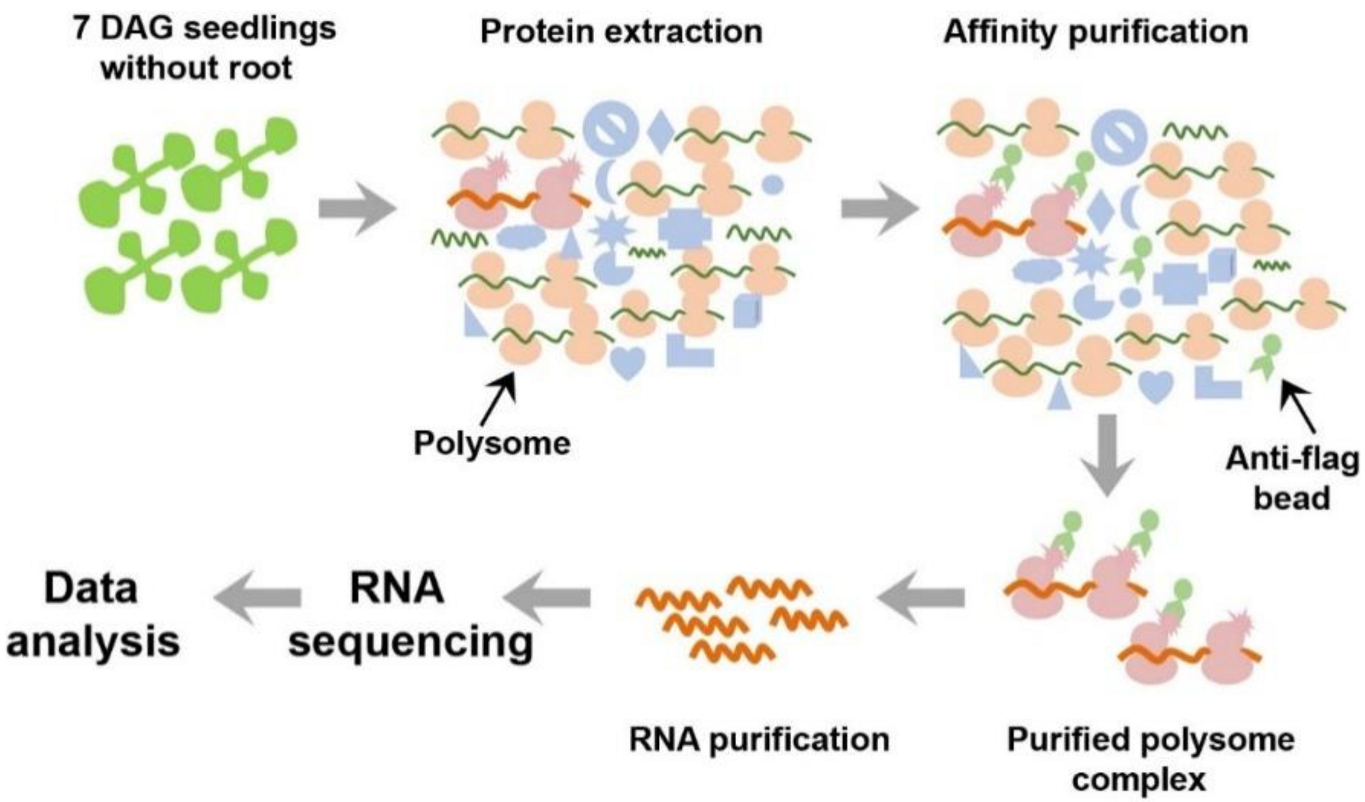

Supplementary Figure 2. Schematic diagram of TRAP-seq.

Shapes represent different kinds of protein, and lines show RNAs. Polysomes and anti-flag beads are indicated. The polysomes in salmon with a star represent the domain specifically expressed ones with HF-epitopes, which can be purified by anti-FLAG beads. Seedlings at 7 DAG (without roots) were ground and HF-tagged polysomes together with translating mRNA were purified by affinity to anti-FLAG beads. The mRNAs, expressed in specific domains, were then extracted and used for library construction and sequencing.

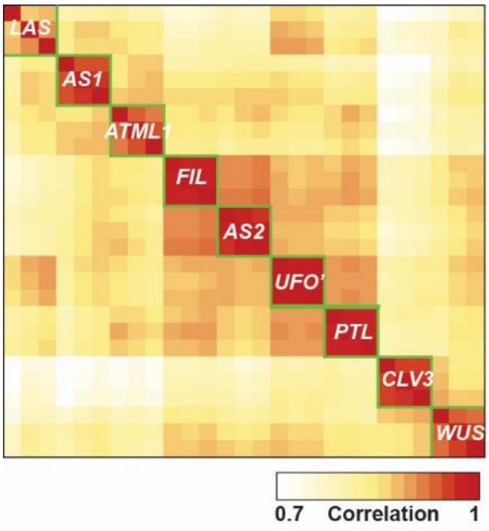

Supplementary Figure 3. Pearson correlation coefficients of translatome data.

Pearson correlation coefficients of translatome data from biological replicates for all profiled tissue domains. The three biological replicates are tightly related to each other and separate from other domains.

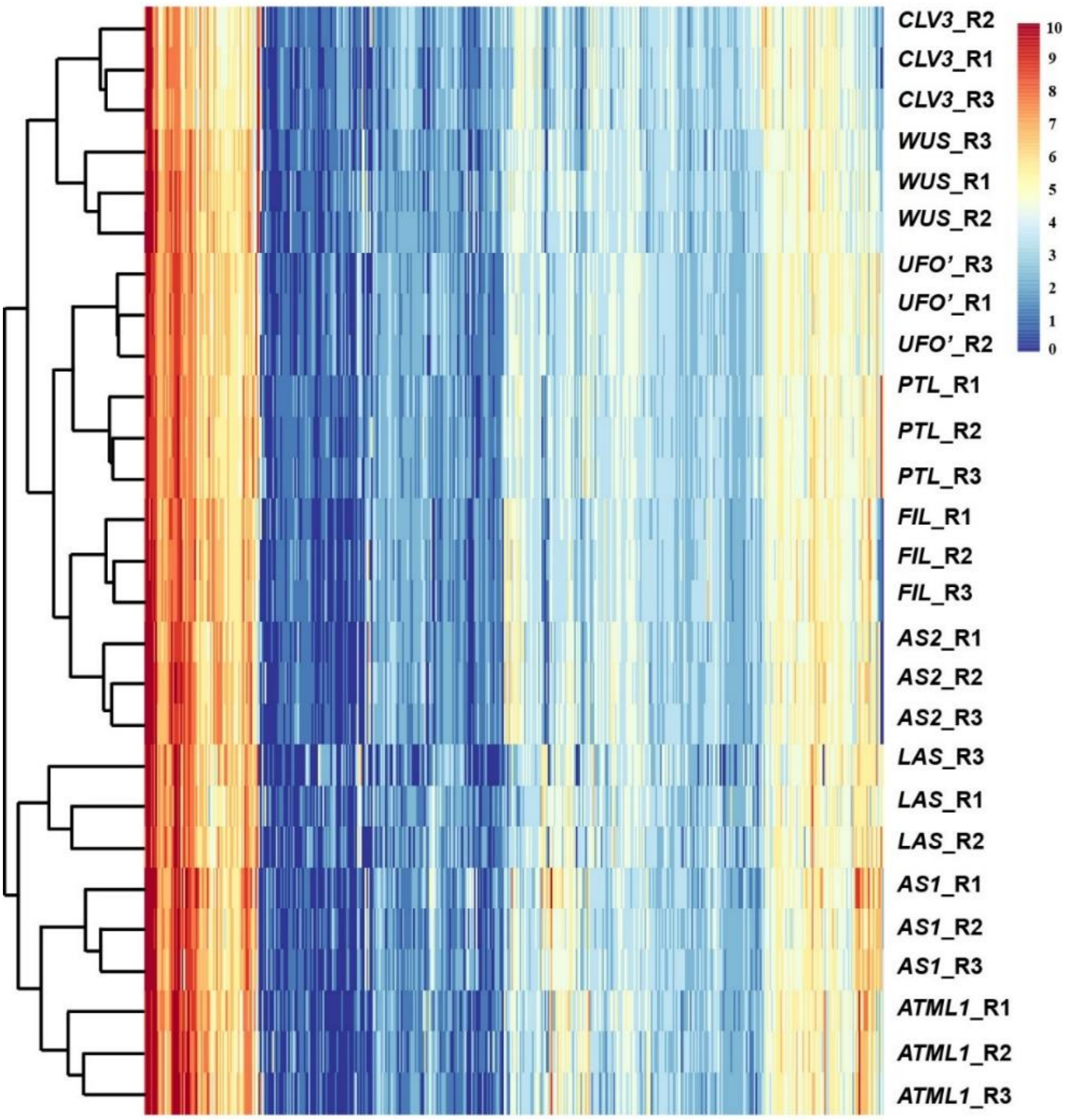

Supplementary Figure 4. Complete hierarchical clustering analysis.

Complete hierarchical clustering analysis of all expressed genes using expression value (Log_{10}RPKM) from biological replicates for different domains, related to Fig. 2. The three biological replicates clustering together indicates the reproducibility of the translatome data.

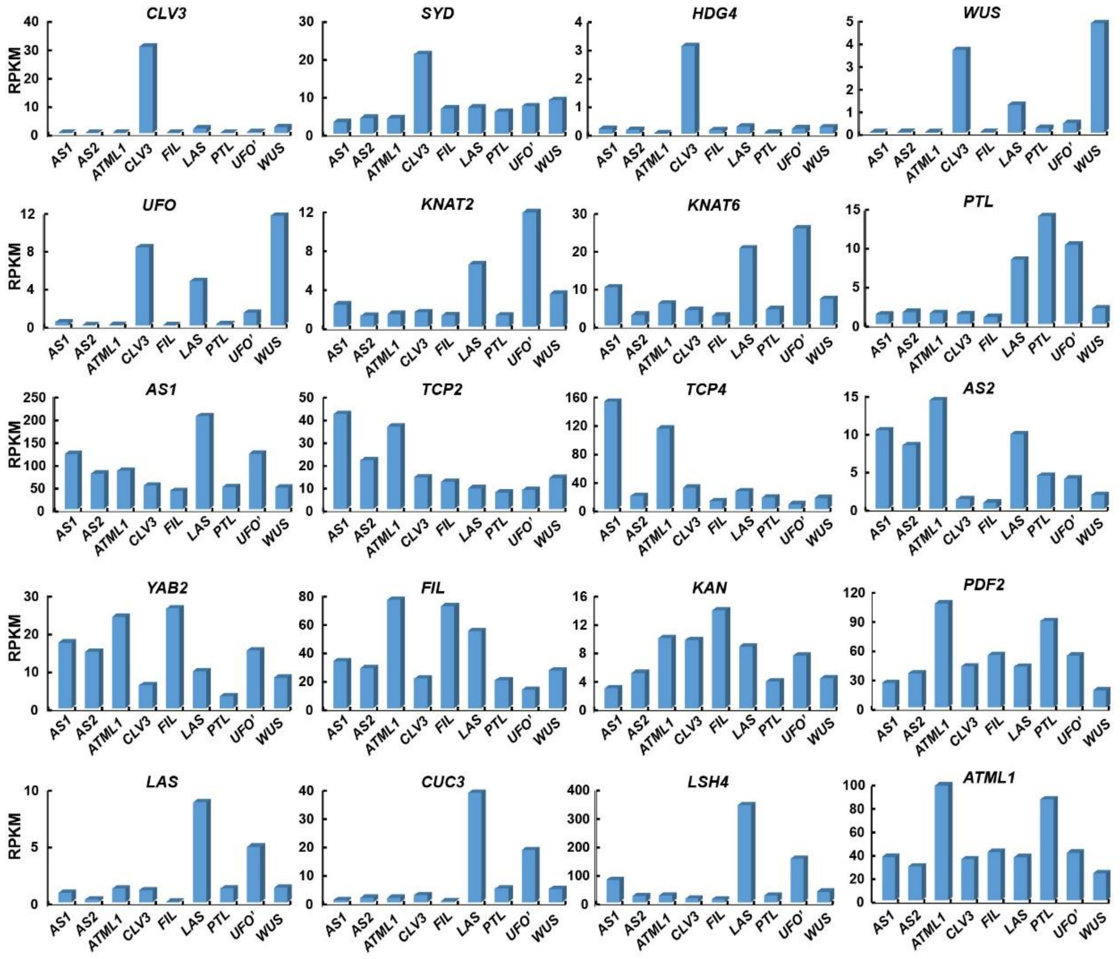

Supplementary Figure 5. Expression patterns of known marker genes, related to Fig. 1b and Supplemental Data 1.

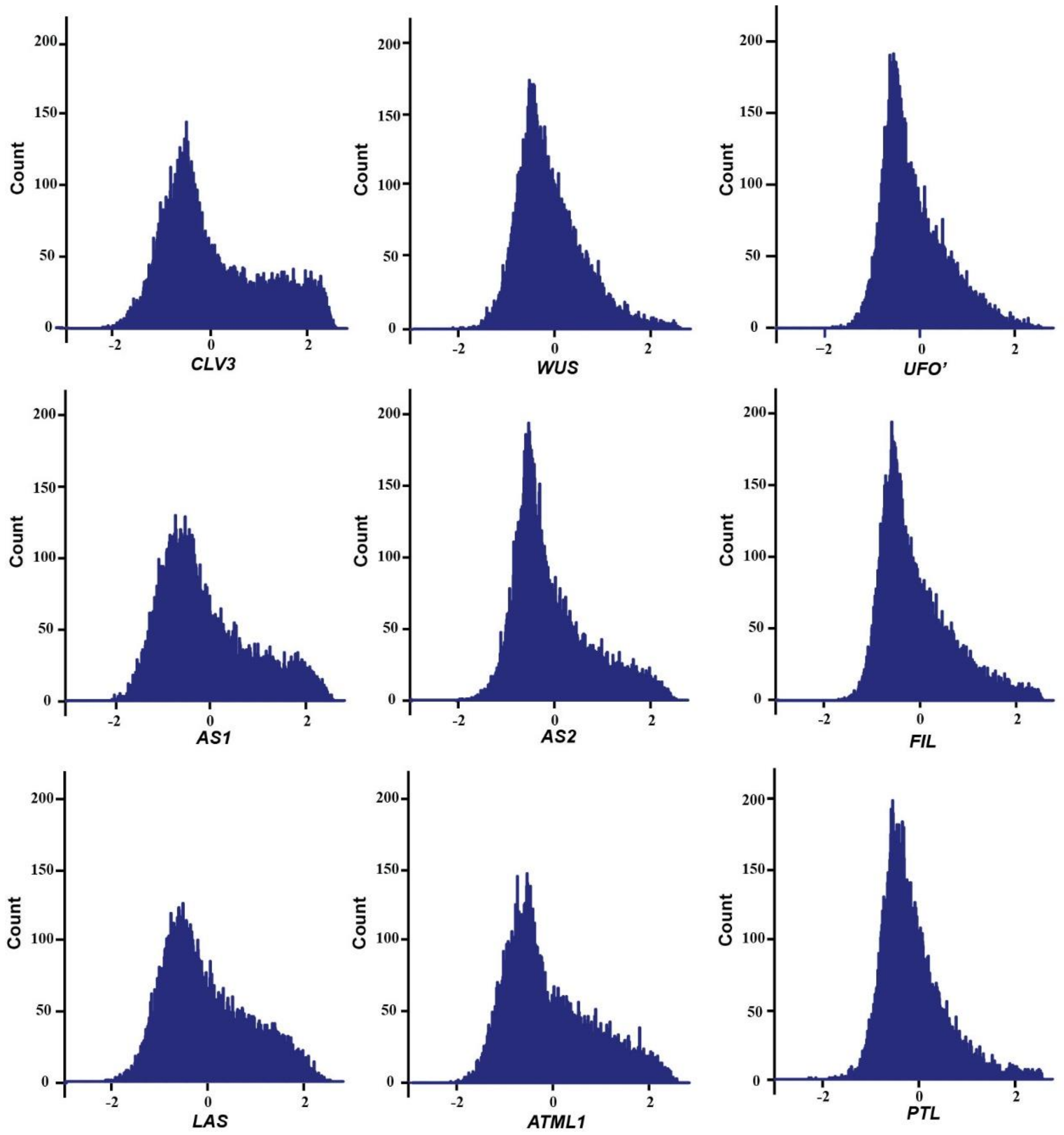

Supplementary Figure 6. Histograms of expression patterns.

Histograms of expression patterns of expressed transcripts represented by the *z*-score. Divergent distributions are displayed with domain-specific pattern, related to Fig. 2.

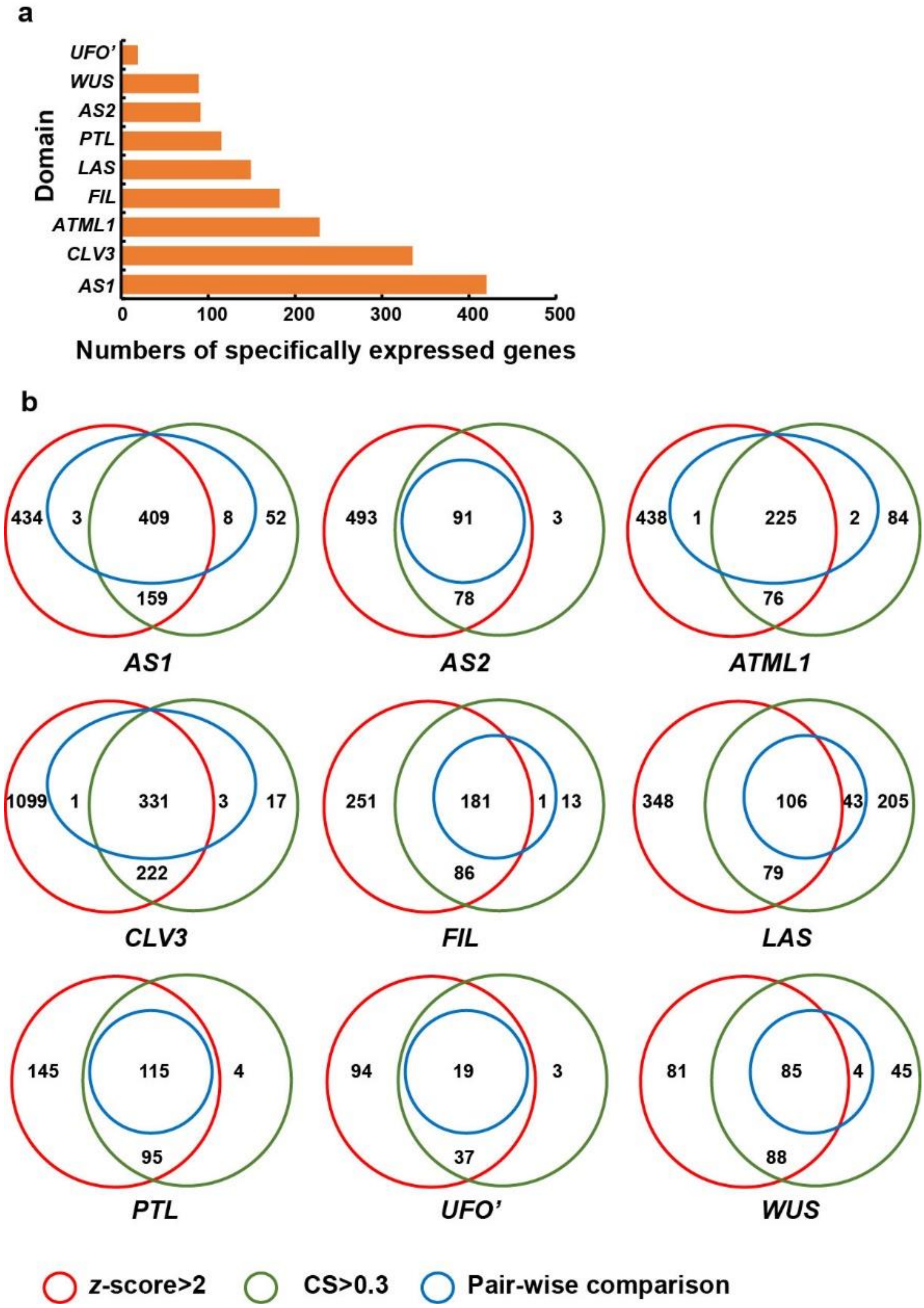

Supplementary Figure 7. Domain specifically expressed genes.

(**a**) Numbers of domain-specifically expressed genes identified by pair-wise comparison. (**b**) Venn diagrams showing domain enriched genes identified by three methods.

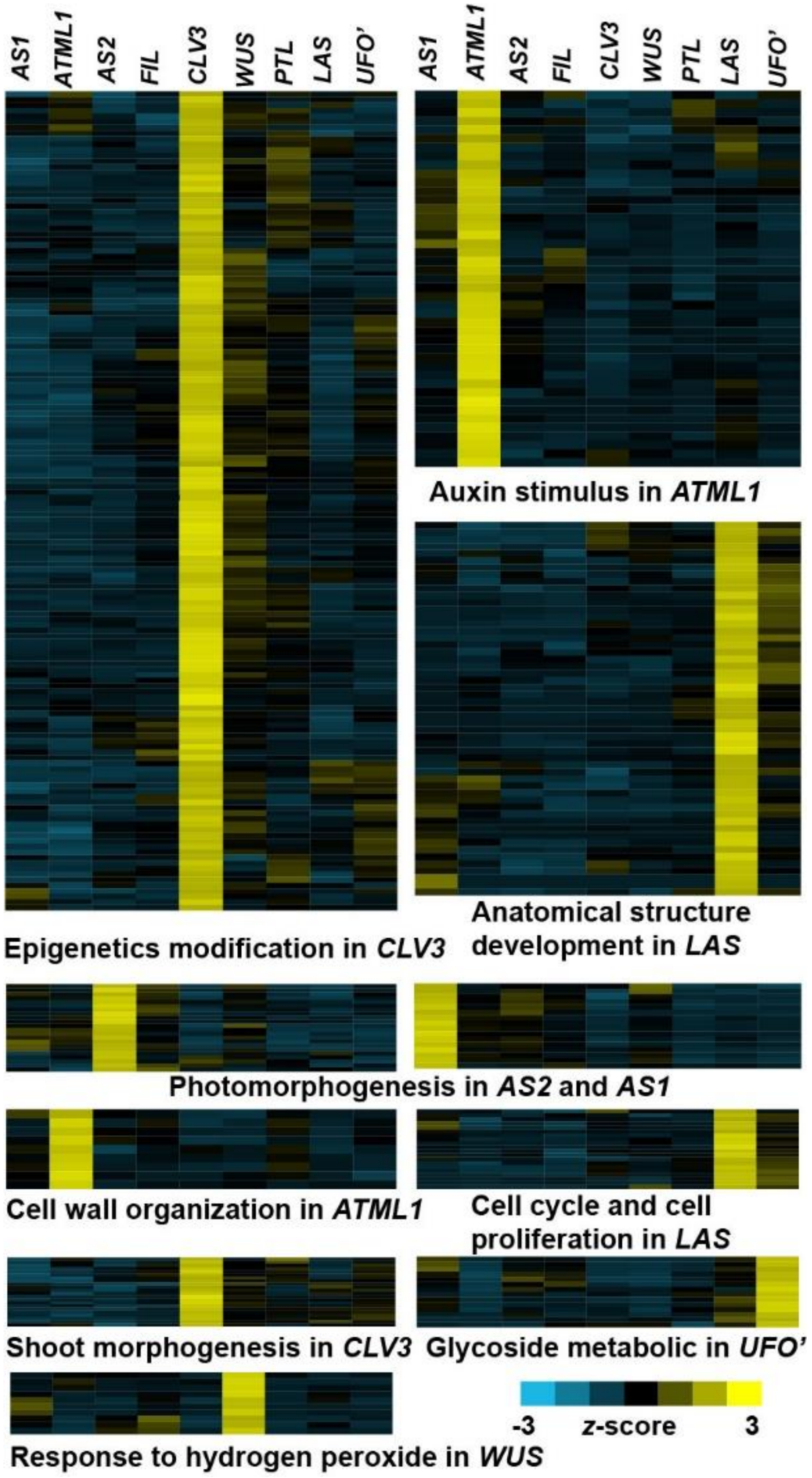

Supplementary Figure 8. GO analysis of domain-enriched genes showing enrichment of selected GO categories.

Expression patterns of enriched genes in each category are depicted in heat maps. Colors represent the relative expression values indicated by *z*-score with low values in blue and high values in yellow.

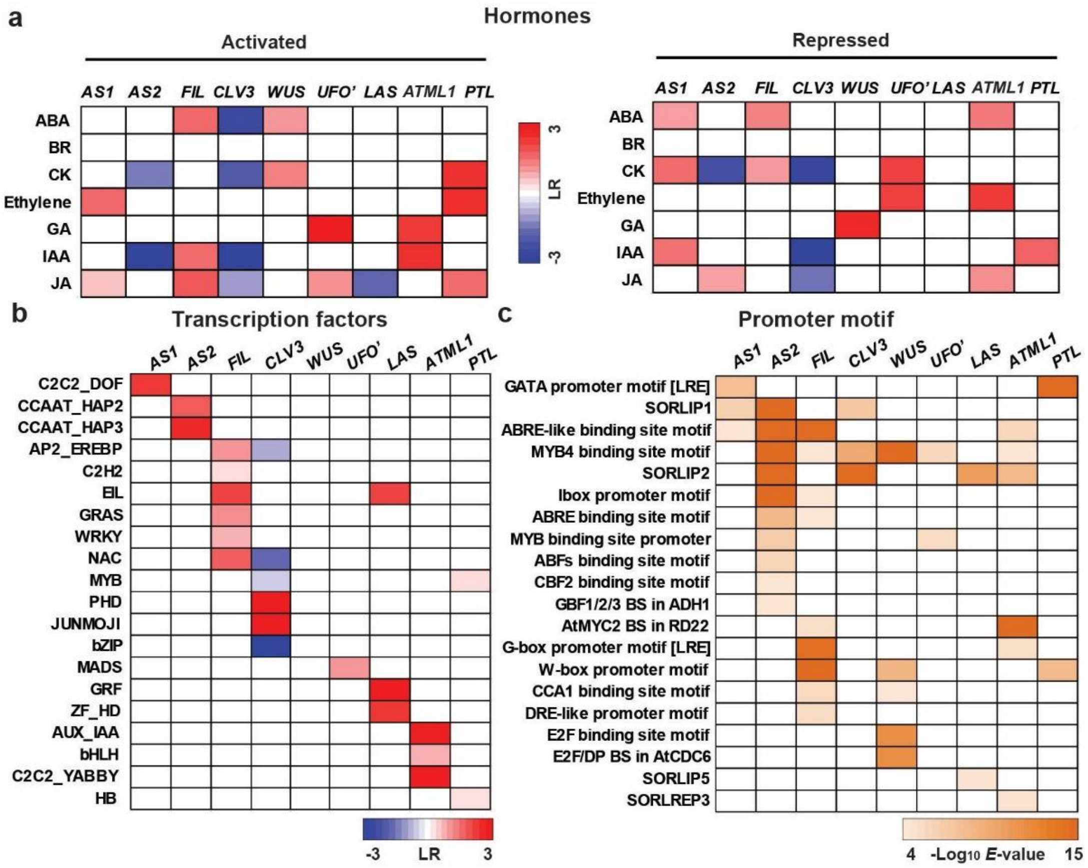

Supplementary Figure 9. Enrichment analysis for different domains.

(**a**) Domain-specific enriched hormone-responsive genes. Red indicates enrichment and blue indicates depletion. Only significantly over-represented categories (FDR adjusted *P*-value < 0.05 by hypergeometric test) are colored. (**b**) Domain-specific enriched transcription factor families. Only significantly over-represented families (FDR adjusted *P*-value < 0.05 by hypergeometric test) are colored. (**c**) Domain-specific enriched of known *cis*-elements in specific domains. Only significantly over-represented motifs (*E*-value < 10^{-4}) are colored.

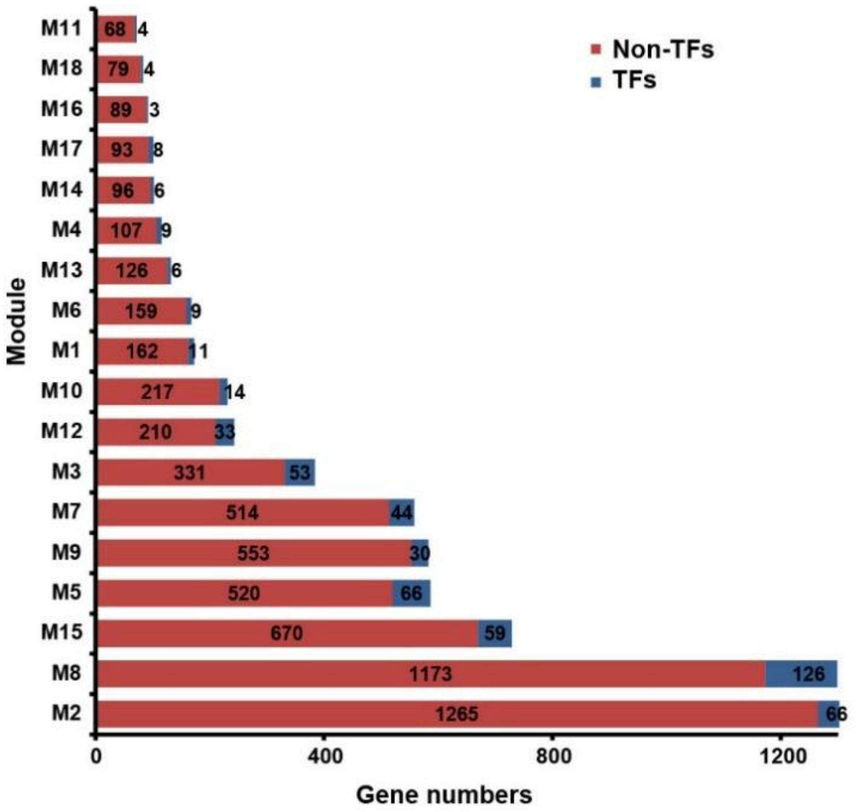

Supplementary Figure 10. Numbers of all genes and transcription factor-encoding genes identified in each GCN module.

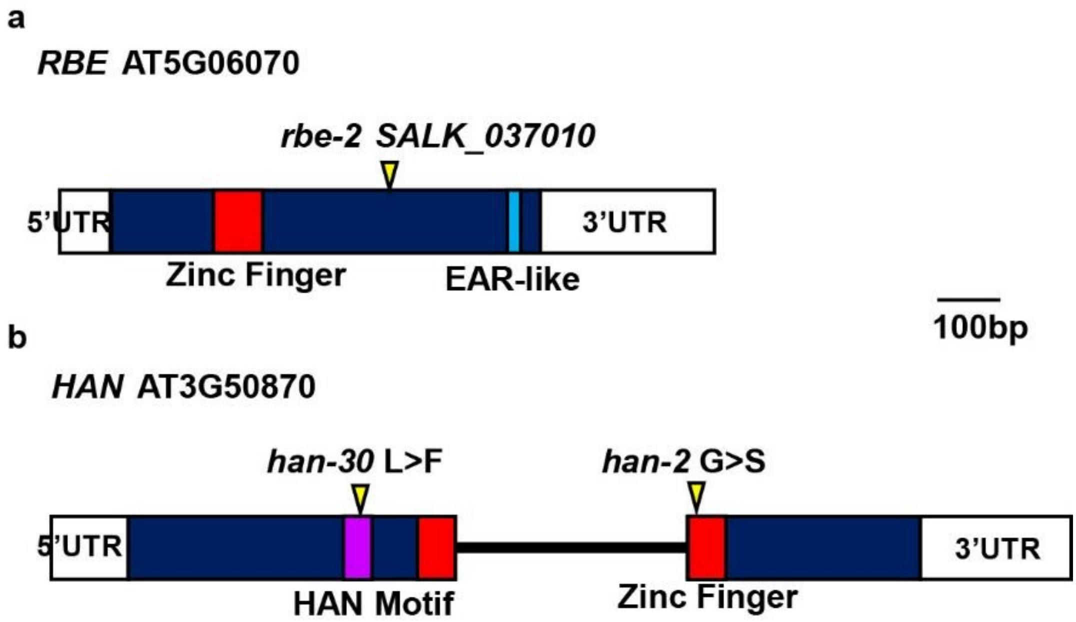

Supplementary Figure 11. The gene structures and mutation sites of *RBE* (**a**) and *HAN* (**b**) genes.

Exons are shown in boxes and introns in lines. Boxes in dark blue represent coding sequences, in which key motifs are displayed in different colors. A T-DNA insertion site of *rbe-2* and two mutation sites of *han-2* and *han-30* are denoted with triangles.

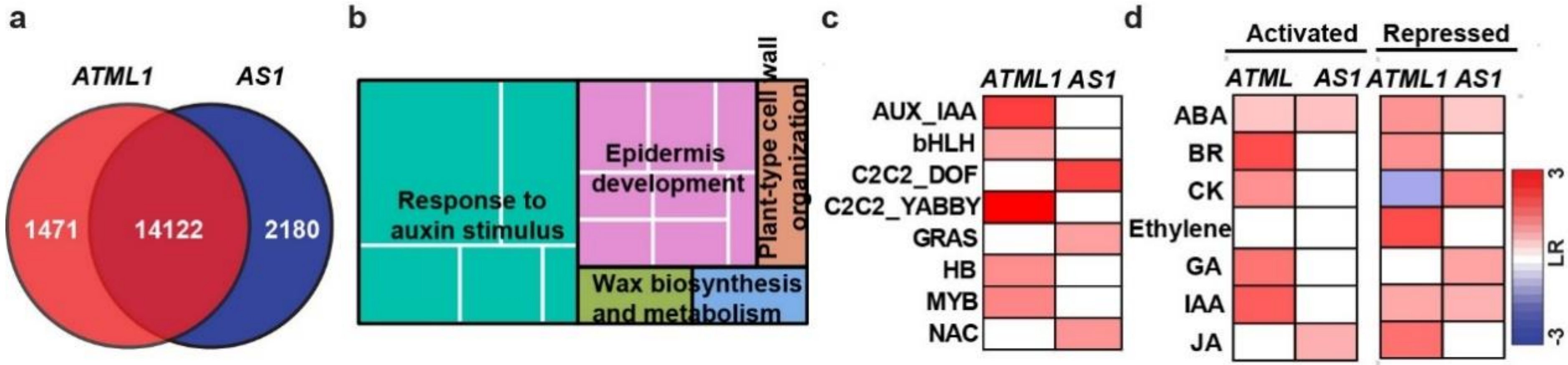

Supplementary Figure 12. Expression pattern comparisons between epidermal domain and leaf primordial domain.

(**a**) Venn diagram of domain-enriched genes that exhibited significant (FC ⩾ two-fold with FDR adjusted *P*-value ⩽ 0.01) up-regulation in epidermal domain (*ATML1*, red) or leaf primordial domain (*AS1*, blue). The number in the middle indicates expressed genes without domain specificity. (**b**) GO analysis of differentially expressed genes in epidermal domain identified significantly enriched gene categories in Cell Wall Organization, Wax Biosynthesis, and Response to Auxin. Aggregate size indicates significance levels for categories by a Yekutieli test with a FDR correction which was summarized by REVIGO. (**c**) Domain-specific enrichment of transcription factors families (FDR adjusted *P*-value ≤ 0.05) between the two domains. (**d**) Domain-specific enrichment of hormone activated and repressed genes (FDR adjusted *P*-value ≤ 0.05) between the two domains.

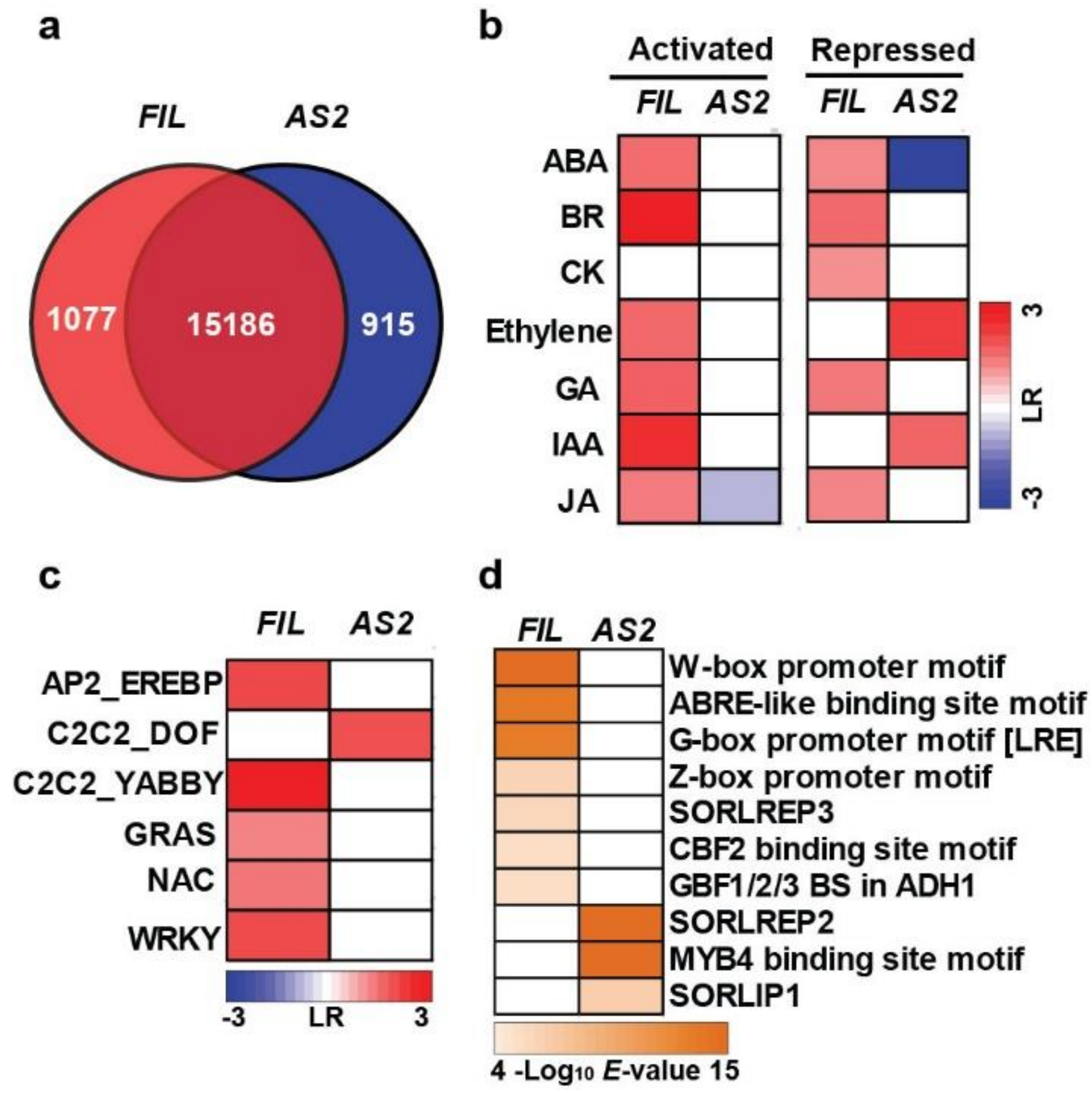

Supplementary Figure 13. Expression pattern comparisons between abaxial and adaxial leaf domains.

(**a**) Venn diagram of cell domain-enriched genes that exhibited significant (FC ≥ two-fold with FDR adjusted *P*-value ≤ 0.01) up-regulation in abaxial (*FIL*, red) or adaxial (*AS2*, blue) leaf domains. The number in the middle indicates expressed genes without domain specificity. (**b**) Domain-specific enrichment of hormone activated and repressed genes (FDR adjusted *P*-value ≤ 0.05) between the two domains. (**c**) Domain-specific enrichment of transcription factor families in the two domains (FDR adjusted *P*-value ≤ 0.05). (**d**) Domain-specific enriched known *cis*-elements in abaxial and adaxial leaf domains. Only significantly overrepresented (*E*-value < 10^{-4}) classes were colored.

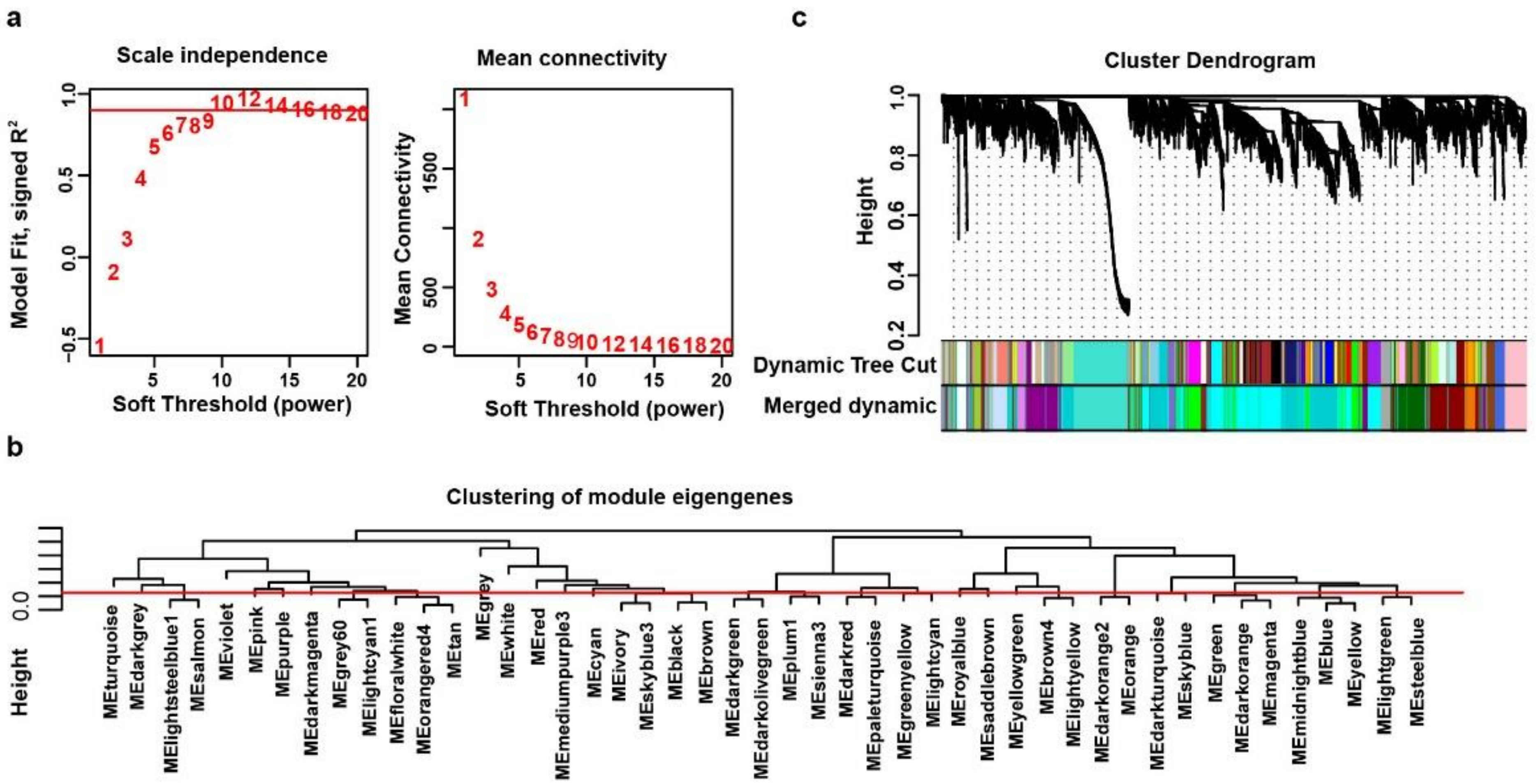

Supplementary Figure 14. Cutoffs and threshold (**a**) and dynamic tree cut (**b** and **c**) used for co-expression network construction by WGCNA.

SUPPLEMENTARY TABLES

Supplementary Table 1. Summary of reads mapping.

Domain	Library Size	Unique Mapped	Multi Mapped	Unique Mapped Ratio
AS2-R1	20,407,777	19,103,058	1,304,719	93.61%
AS2-R2	20,948,752	19,550,404	1,398,348	93.32%
AS2-R3	21,461,115	20,106,508	1,354,607	93.69%
ATML1-R1	45,740,291	43,416,745	2,323,546	94.92%
ATML1-R2	19,578,140	18,306,700	1,271,440	93.51%
ATML1-R3	12,341,409	11,609,417	731,992	94.07%
CLV3-R1	13,089,870	12,208,916	880,954	93.27%
CLV3-R2	18,068,682	16,774,903	1,293,779	92.84%
CLV3-R3	21,013,986	19,786,117	1,227,869	94.16%
FIL-R1	20,480,184	19,470,951	1,009,233	95.07%
FIL-R2	21,343,660	20,334,273	1,009,387	95.27%
FIL-R3	23,568,618	22,415,861	1,152,757	95.11%
PTL-R1	20,676,628	19,655,125	1,021,503	95.06%
PTL-R2	19,327,737	18,332,593	995,144	94.85%
PTL-R3	22,624,393	21,468,061	1,156,332	94.89%
UFO'-R1	23,725,720	22,727,306	998,414	95.79%
UFO'-R2	24,694,401	23,705,747	988,654	96.00%
UFO'-R3	29,554,451	28,327,045	1,227,406	95.85%
WUS-R1	18,284,149	17,631,889	652,260	96.43%
WUS-R2	18,487,847	17,864,222	623,625	96.63%
WUS-R3	16,726,233	16,137,075	589,158	96.48%

Supplementary Table 2. Gene Ontology terms of leaf adaxial enriched genes.

GO term	Ontology	Description	Number in input list	Number in BG/Ref	FDR
GO:0044085	Biological Process	Cellular component biogenesis	66	433	1.54E-15
GO:0009058	Biological Process	Biosynthetic process	198	2933	2.66E-06
GO:0044249	Biological Process	Cellular Biosynthetic process	196	2783	2.00E-07
GO:0034645	Biological Process	Cellular macromolecule biosynthetic process	162	1920	1.61E-11
GO:0009853	Biological Process	Photorespiration	7	34	3.99E-02
GO:0009059	Biological Process	Macromolecule biosynthetic process	163	1937	1.61E-11
GO:0015979	Biological Process	Photosynthesis	21	107	1.16E-06
GO:0006091	Biological Process	Generation of precursor metabolites and energy	19	173	1.77E-02
GO:0019538	Biological Process	Protein metabolic process	132	2139	1.05E-02
GO:0019684	Biological Process	Photosynthesis, light reaction	12	63	1.71E-03
GO:0010467	Biological Process	Gene expression	162	2059	2.48E-09
GO:0022900	Biological Process	Electron transport chain	10	52	7.31E-03
GO:0045454	Biological Process	photosynthetic electron transport in photosystem I	6	13	1.06E-03
GO:0022613	Biological Process	Ribonucleprotein complex biogenesis	45	179	6.71E-19
GO:0042254	Biological Process	Ribosome biogenesis	45	173	2.25E-19
GO:0009834	Biological Process	Secondary cell wall biogenesis	5	14	1.77E-02
GO:0034622	Biological Process	Cellular macromolecular complex assembly	16	143	3.99E-02
GO:0006412	Biological Process	Translation	102	505	9.29E-34
GO:0044267	Biological Process	Cellular protein metabolic process	127	1853	2.06E-04
GO:0019725	Biological Process	Photosynthetic electron transport chain	7	27	1.08E-02

Supplementary Table 3. Transgenic and mutant lines used in this work.

Lines	Sources	Background	Expression pattern
pAS2::LhG4	This work	*Ler*	Leaf adaxial
pATML1::LhG4	From Thomas Laux[1]	*Ler*	L1 layer
pCLV3::HF-RPL18	This work	*Ler*	Central zone
pFIL::LhG4	From Yuval Eshed[2]	*Ler*	Leaf abaxial
pOp::GFP-ER	From Yuval Eshed[2]	*Ler*	
pOp::HF-RPL18 35S::AP1-GR ap1-1 cal1	This lab[3]	*Ler*	
pPTL::LhG4	From David Smyth[4]	Col-0	Leaf margin
pUFO::LhG4	This work	*Ler*	Peripheral zone
pWUS::LhG4	From Thomas Laux[5]	*Ler*	Organization center
rbe-2	From Tengbo Huang[6]	Col-0	
han-2	From Elliot M. Meyerowitz[7]	Col-0	
han-30	From Hirokazu Tsukaya[8]	Col-0	

Supplementary Table 4. Primers used in this work.

Primer name	Primer sequence	Usage
pAS2-F-SalI	gtcgacTAGGGCCCGAAATGTGGAAA	Construction of *pAS2::LhG4*
pAS2-R-PstI	aactgcagTGTTGAAGAAGATGCCAT	Construction of *pAS2::LhG4*
pCLV3-FP-SalI	aaagtcgacCATATCCATAAAATTAATCGAATT	Construction of *pCLV3::HF-RPL18*
pCLV3-RP-PstI	aaactgcagTTTTAGAGAGAAAGTGACTGAG	Construction of *pCLV3::HF-RPL18*
tCLV3-FP-ClaI	aaaatcgatCCTAATCTCTTGTTGCTTTAAA	Construction of *pCLV3::HF-RPL18*
tCLV3-RP-BamHI	aaaggatccCTACAATGTTGATTACTTTGGTATA	Construction of *pCLV3::HF-RPL18*
pCLV3-iden-F	CTGTCCCCTTCTCATTTC	Sequencing
HF-RPL18-R	AACCTTGAATCCACGACTCTTC	Sequencing
pUFO-FP	atgtcgacCGACACGCAACGACTCCTATCG	Construction of *pUFO::LhG4*
pUFO-RP	gggtaccTTTAGCTGAAAAATGAAAAGATTT	Construction of *pUFO::LhG4*
SALK_037010-RP	CTTTATTTTGGCCGTTTAGGG	Genotyping of *rbe-2*
SALK_037010-LP	GGAGTTCACATGGAATACCAGAC	Genotyping of *rbe-2*
LB1.3	ATTTTGCCGATTTCGGAAC	Genotyping of *rbe-2*
han-30-FP	ATGCAGACTCCGTACACTAC	Genotyping of *han-30*
han-30-RP	TTGGTGTGAATCAAGTCCCAAA	Genotyping of *han-30*
han-2-FP	tttgcagTCCCTATGCAACGC	Genotyping of *han-2*
han-2-RP	CTATCCGCTACATTAAGCCTC	Genotyping of *han-2*

Supplementary References

1. Lenhard, M. & Laux, T. Stem cell homeostasis in the *Arabidopsis* shoot meristem is regulated by intercellular movement of CLAVATA3 and its sequestration by CLAVATA1. *Development* **130**, 3163-3173 (2003).
2. Goldshmidt, A., Alvarez, J.P., Bowman, J.L. & Eshed, Y. Signals derived from YABBY gene activities in organ primordia regulate growth and partitioning of Arabidopsis shoot apical meristems. *Plant Cell* **20**, 1217-1230 (2008).
3. Jiao, Y. & Meyerowitz, E.M. Cell-type specific analysis of translating RNAs in developing flowers reveals new levels of control. *Mol Syst Biol* **6**, 419 (2010).
4. Brewer, P.B. et al. *PETAL LOSS*, a trihelix transcription factor gene, regulates perianth architecture in the *Arabidopsis* flower. *Development* **131**, 4035-4045 (2004).
5. Gross-Hardt, R., Lenhard, M. & Laux, T. *WUSCHEL* signaling functions in interregional communication during *Arabidopsis* ovule development. *Genes & development* **16**, 1129-1138 (2002).
6. Huang, T., Lopez-Giraldez, F., Townsend, J.P. & Irish, V.F. RBE controls microRNA164 expression to effect floral organogenesis. *Development* **139**, 2161-2169 (2012).
7. Zhang, X. et al. Transcription repressor HANABA TARANU controls flower development by integrating the actions of multiple hormones, floral organ specification genes, and GATA3 family genes in *Arabidopsis*. *Plant Cell* **25**, 83-101 (2013).
8. Kanei, M., Horiguchi, G. & Tsukaya, H. Stable establishment of cotyledon identity during embryogenesis in *Arabidopsis* by *ANGUSTIFOLIA3* and *HANABA TARANU*. *Development* **139**, 2436-2446 (2012).
9. Sessions, A., Weigel, D., & Yanofsky, M.F. The *Arabidopsis thaliana MERISTEM LAYER 1* promoter specifies epidermal expression in meristems and young primordia. *Plant J.* **20**, 259-263 (1999).
10. Abe, M., Katsumata, H., Komeda, Y., & Takahashi, T. Regulation of shoot

epidermal cell differentiation by a pair of homeodomain proteins in *Arabidopsis*. *Development* **130**, 635-643 (2003).

11. Siegfried, K.R.*et al.* Members of the *YABBY* gene family specify abaxial cell fate in *Arabidopsis*. *Development* **126**, 4117-4128 (1999).
12. Sawa, S.*et al. FILAMENTOUS FLOWER*, a meristem and organ identity gene of *Arabidopsis*, encodes a protein with a zinc finger and HMG-related domains. *Genes Dev.* **13**, 1079-1088 (1999).
13. Kerstetter, R.A., Bollman, K., Taylor, R.A., Bomblies, K., & Poethig, R.S. KANADI regulates organ polarity in *Arabidopsis*. *Nature* **411**, 706-709 (2001).
14. Palatnik, J.F. *et al.* Control of leaf morphogenesis by microRNAs. *Nature* **425**, 257-263 (2003).
15. Iwakawa, H.*et al.* Expression of the *ASYMMETRIC LEAVES2* gene in the adaxial domain of *Arabidopsis* leaves represses cell proliferation in this domain and is critical for the development of properly expanded leaves. *Plant J.* **51**, 173-184 (2007).
16. Eshed, Y., Baum, S.F., Perea, J.V., & Bowman, J.L. Establishment of polarity in lateral organs of plants. *Curr. Biol.* **11**, 1251-1260 (2001).
17. Greb, T. *et al.* Molecular analysis of the *LATERAL SUPPRESSOR* gene in *Arabidopsis* reveals a conserved control mechanism for axillary meristem formation. *Genes Dev.* **17**, 1175-1187 (2003).
18. Vroemen, C.W., Mordhorst, A.P., Albrecht, C., Kwaaitaal, M.A., & de Vries, S.C. The *CUP-SHAPED COTYLEDON3* gene is required for boundary and shoot meristem formation in *Arabidopsis*. *Plant Cell* **15**, 1563-1577 (2003).
19. Takeda, S. *et al.* CUP-SHAPED COTYLEDON1 transcription factor activates the expression of *LSH4* and *LSH3*, two members of the ALOG gene family, in shoot organ boundary cells. *Plant J.* **66**, 1066-1077 (2011).
20. Belles-Boix, E. *et al. KNAT6*: an Arabidopsis homeobox gene involved in meristem activity and organ separation. *Plant Cell* **18**, 1900-1907 (2006).
21. Yadav, R.K., Tavakkoli, M., Xie, M., Girke, T., & Reddy, G.V. (2014). A high-resolution gene expression map of the *Arabidopsis* shoot meristem stem cell

niche. *Development* **141**, 2735-2744.

22. Brand, U., Fletcher, J.C., Hobe, M., Meyerowitz, E.M., & Simon, R. Dependence of stem cell fate in *Arabidopsis* on a feedback loop regulated by *CLV3* activity. *Science* **289**, 617-619 (2000).
23. Lee, I., Wolfe, D.S., Nilsson, O., & Weigel, D. A *LEAFY* co-regulator encoded by *UNUSUAL FLORAL ORGANS*. *Curr. Biol.* **7**, 95-104 (1997).
24. Mayer, K.F. *et al.* Role of *WUSCHEL* in regulating stem cell fate in the *Arabidopsis* shoot meristem. *Cell* **95**, 805-815 (1998).
