# Supplementary material for: A gene expression map of shoot domains reveals regulatory mechanisms
Source: Nat Commun. 2019 Jan 11;10:141. doi: 10.1038/s41467-018-08083-z (PMC6329838; doi:10.1038/s41467-018-08083-z)
Supplement: Supplementary file 3 — Description of Additional Supplementary Files [file 41467_2018_8083_MOESM3_ESM.pdf]

File Name: Supplementary Dataset 1

Description: The list of marker genes indicated in Figure 1b.

File Name: Supplementary Dataset 2

Description: Domain specifically expressed genes by pair-wise comparison.

File Name: Supplementary Dataset 3

Description: Gene Ontology terms of domain specifically expressed genes.

File Name: Supplementary Dataset 4

Description: Domain-enriched genes.

File Name: Supplementary Dataset 5

Description: Expressed and domain-enriched LncRNAs.

File Name: Supplementary Dataset 6

Description: Domain-enriched Pseudogenes.

File Name: Supplementary Dataset 7

Description: List of genes in GCN module M12.

File Name: Supplementary Dataset 8

Description: List of genes in GCN module M5.

File Name: Supplementary Dataset 9

Description: Differential expressed genes among SAM cell types.

File Name: Supplementary Dataset 10

Description: Differential expressed genes in epidermis cell type.

File Name: Supplementary Dataset 11

Description: Differential expressed genes in adaxial/abaxial leaf cells.

File Name: Supplementary Dataset 12

Description: Lists of hormone-responsive genes used for meta analysis.

File Name: Supplementary Data 13.

Description: Lists of transcription factor genes used for meta analysis.
